# ‘Dusty core disease’ (DuCD): expanding morphological spectrum of *RYR1* recessive myopathies

**DOI:** 10.1186/s40478-018-0655-5

**Published:** 2019-01-05

**Authors:** Matteo Garibaldi, John Rendu, Julie Brocard, Emmanuelle Lacene, Julien Fauré, Guy Brochier, Maud Beuvin, Clemence Labasse, Angeline Madelaine, Edoardo Malfatti, Jorge Alfredo Bevilacqua, Fabiana Lubieniecki, Soledad Monges, Ana Lia Taratuto, Jocelyn Laporte, Isabelle Marty, Giovanni Antonini, Norma Beatriz Romero

**Affiliations:** 1 Neuromuscular Morphology Unit, Myology Institute, Groupe Hospitalier Universitaire La Pitié-Salpêtrière, Paris, France; 2 Unit of Neuromuscular Diseases, Neuromuscular Disease Centre, Department of Neurology Mental Health and Sensory Organs (NESMOS), Faculty of Medicine and Psychology, SAPIENZA University of Rome, Sant’Andrea Hospital, Via di Grottarossa 1035-1039, 00189 Rome, Italy; 3 Centre Hospitalier Universitaire de Grenoble Alpes, Biochimie Génétique et Moléculaire, Grenoble, France; 4 Grenoble Institut des Neurosciences- Inserm U1216 – UGA, Grenoble, France; 5 Sorbonne Universités UPMC Univ Paris 06- Inserm UMRS974, Center of Research in Myology, Institut de Myologie, Centre de Référence Maladies Neuromusculaire Paris-Est-Ile de France, Groupe Hospitalier Pitié-Salpêtrière, Paris, France; 6 Service Neurologie Médicale, Centre de Référence Maladies Neuromusculaire Paris-Est-Ile de France, CHU Raymond-Poincaré Paris Ouest, Garches, France; 7 U1179 UVSQ-INSERM Handicap Neuromusculaire: Physiologie, Biothérapie et Pharmacologie appliquées, UFR des sciences de la santé Simone Veil, Université Versailles-Saint-Quentin-en-Yvelines, Versailles, France; 8 Neuromuscular Unit, Department of Neurology and Neurosurgery, University of Chile Clinical Hospital, Santiago, Chile; 9 Department of Anatomy and Legal Medicine, Faculty of Medicine, University of Chile, Santiago, Chile; 10 Servicio de Neurología y Servicio de Patologia, Hospital de Pediatría Garrahan, Buenos Aires, Argentina; 11 Neuropathology, Foundation for Neurological Research (FLENI), Buenos Aires, Argentina; 12 Institut de Génétique et de Biologie Moléculaire et Cellulaire (IGBMC), 1, rue Laurent Fries, BP 10142, 67404 Illkirch, France; 13 INSERM U1258, 67404 Illkirch, France; 14 CNRS, UMR7104, 67404 Illkirch, France; 15 Université de Strasbourg, 67404 Illkirch, France

**Keywords:** RYR1 recessive, Dusty Core Disease, Central Core Disease, Congenital Myopathy, Centronuclear myopathy, Ryanodine receptor

## Abstract

Several morphological phenotypes have been associated to *RYR1*-recessive myopathies. We recharacterized the *RYR1*-recessive morphological spectrum by a large monocentric study performed on 54 muscle biopsies from a large cohort of 48 genetically confirmed patients, using histoenzymology, immunohistochemistry, and ultrastructural studies. We also analysed the level of RyR1 expression in patients’ muscle biopsies. We defined “dusty cores” the irregular areas of myofibrillar disorganisation characterised by a reddish-purple granular material deposition with uneven oxidative stain and devoid of ATPase activity, which represent the characteristic lesion in muscle biopsy in 54% of patients. We named Dusty Core Disease (DuCD) the corresponding entity of congenital myopathy. Dusty cores had peculiar histological and ultrastructural characteristics compared to the other core diseases. DuCD muscle biopsies also showed nuclear centralization and type1 fibre predominance. Dusty cores were not observed in other core myopathies and centronuclear myopathies. The other morphological groups in our cohort of patients were: Central Core (CCD: 21%), Core-Rod (C&R:15%) and Type1 predominance “plus” (T1P+:10%). DuCD group was associated to an earlier disease onset, a more severe clinical phenotype and a lowest level of RyR1 expression in muscle, compared to the other groups. Variants located in the bridge solenoid and the pore domains were more frequent in DuCD patients. In conclusion, DuCD is the most frequent histopathological presentation of *RYR1*-recessive myopathies. Dusty cores represent the unifying morphological lesion among the DuCD pathology spectrum and are the morphological hallmark for the recessive form of disease.

**Electronic supplementary material:**

The online version of this article (10.1186/s40478-018-0655-5) contains supplementary material, which is available to authorized users.

## Introduction

The *RYR1* gene encodes the ryanodine receptor channel 1 (RyR1), a sarcoplasmic reticulum (SR) calcium channel involved in excitation-contraction coupling through interaction with the dihydropyridine receptor (DHPR) in the T-tubule. *RYR1*-mutations are the most common cause of congenital myopathies with cores [14]. Variable clinical and histological phenotypes have been described in both dominant and recessive forms [36]. Dominant mutations in N-terminal and central regions (MHS/CCD1 and MHS/CCD2 domains respectively) are classically associated to Malignant Hyperthermia Susceptibility (MHS) trait, whereas dominant mutations in C-terminal region (MHS/CCD3) are more likely associated to Central Core Disease (CCD) [40]. Moreover, heterozygous variants have been previously reported in core-rod myopathy [12, 27, 33] and more recently in Exertional Rhabdomyolysis (ERM) [9] and late-onset axial myopathy [15, 23].

By contrast, recessive forms of disease manifest a wider spectrum of clinical and histological presentations. Even if *RYR1*-recessive patients could present a clinical picture similar to the dominant one, including an early onset, non-progressive, proximal muscle weakness, other peculiar clinical phenotypes of recessive forms are characterized by a more diffuse muscle weakness, ocular involvement with ptosis and/or ophthalmoplegia, and a severe bulbar and respiratory muscle weakness [19, 20, 35]. Nevertheless, severe clinical presentations and foetal akinesia syndrome have been reported in both dominant and recessive forms [5, 12, 31].

Concerning morphology, the phenotypic variability of histopathological findings in recessive forms increases even more, including CCD [10, 16, 31, 41], Multiminicore Disease (MmD) [11, 18, 25], Congenital Fibre Type Disproportion (CFTD) [7] and Centronuclear Myopathy (CNM) [1, 17, 37]. Our group also described seven *RYR1-*recessive patients showing prominent nuclear internalization and large areas of myofibrillar disorganization [4]. Despite this, in the largest review of 106 *RYR1*-recessive cases, up to 40% of patients did not fill these categories and were classified as Atypical Core Myopathy or different nonspecific histopathological groups [2].

Recessive cases also present an increasing complexity, given the large amount of novel variants detected by the massive sequencing technologies, which makes the genotype-phenotype correlation more challenging. In general, hypomorphic variants (non-sense, frameshift, splice site variants), reducing or abolishing RyR1 production, seem to be more frequent in recessive forms and are associated to a more severe clinical presentation, compared to non-hypomorphic (missense, in-frame ins/del) variants [26, 40]. Nevertheless, up to now, no association between clinical severity and histopathological findings has been found [2].

We report an extensive monocentric analysis of 54 muscle biopsies from a large cohort of 48 *RYR1*-recessive patients to have a homogenous interpretation of morphological findings with the aim to find a closer correlation between morphology, clinical phenotype and genetic background. Here, we describe and define the “dusty core fibres” as the characteristic and unifying morphological feature present in most of *RYR1*-recesssive biopsies and we correlate the RyR1 expression level in patients’ muscle biopsies with clinical and morphological features.

## Materials and methods

### Patients’ sample selection

All the muscle biopsies were analysed at the Neuromuscular Morphology Unit of Myology Institute, in Paris. More than 11000 muscle biopsies collected between 1977 and 2015 were screened. Two hundred and thirty belonged to patients with *RYR1*-related myopathy, of whom 154 had dominant inheritance and 76 were confirmed or suspected to be recessive. In 13 cases the second variant was not found or was not surely pathogenetic (likely pathogenetic, VUS or likely benign). Among the 63 confirmed *RYR1*-recessive patients, 15 cases have been excluded because of insufficient data (uncomplete clinical and/or morphological data and deteriorated muscle sample for re-analysis). Finally, our study cohort consisted on 48 confirmed *RYR1*-recessive patients (20 male and 28 female) from 45 unrelated families. Clinical data were obtained from a full revision of all available medical records up to the last clinical examination in all enrolled patients. Disease onset (considered as the first reported clinical sign referred to disease, including perinatal problems or delayed motor milestones), weakness distribution (proximal/distal limb muscles, axial, facial, ocular/extraocular and bulbar muscles), contractures, spinal deformities and dysmorphisms, respiratory and cardiac involvement were considered. Clinical evaluations were performed by different clinicians over 40 years, thus we retrospectively classified patients in three main groups of clinical severity: mild (late-adult onset, walkers, mild muscle weakness, minimal or absent ocular, facial, bulbar or respiratory involvement), moderate (early onset, non-progressive, proximal or diffuse muscle weakness, associate with mild dysmorphism, spine deformities or contractures) and severe (muscle hypotonia at birth, feeding difficulties, severe respiratory involvement requiring ventilation, contractures and/or spinal deformities, diffuse muscle weakness with facial and ocular involvement).

### Genetic analysis

Total RNA was extracted from each skeletal muscle sample lysed in Trizol reagent (Invitrogen, Life Technologies SAS). Complementary DNA was synthesized from 500 to 750 ng of total RNA using 0.5 μl of Transcriptor (Roche) and 0.3 lg of oligo-dT as described [26]. Seven overlapping PCR amplification spanning the entire *RYR1* sequence were performed. Each fragment was sequenced as previously described [26, 28]. Each variation was confirmed on DNA sample and on both paternal and maternal DNA sample to establish the transmission. Each variant was analysed by Variant effect Predictors to obtain the different prediction score such as CADD, SIFT, Polyphen and gnomAD exome and gnomAD Genome database frequency. To better assess the functional effect of each missense variation, 3D analysis was performed on Yasara sofware [21]. Due to the large size of the *RYR1* gene, we choose not to use the total RyR1 protein structure already described (5gl1/5taz) [3, 8] in the first-round analysis via FoldX prediction. We choose to split the structure in 5 parts spanning the whole human RYR1 structures (amino acid 1 to 627, 628 to 1656, 1657 to 2144, 2145 to 3613 and 3614 to 5038). Then, the sequences were submitted in I-TASSER server [39] to obtain “friendly” usable RyR1 structure. Each structure prediction was matched with the RyR1 global structure (5gl1 and 5taz) [3, 8]. Delta G variations were calculated to estimate protein stability. For delta G variation <0.5 kcal/mol, meaning no destabilization, study of the whole structure was realized (5gl1/5taz) [3, 8]. For ACMG classification, Intervar was used with recessive transmission correction [22].

### Histological study

Histoenzymological analysis was conducted on 54 muscle biopsies (4 patients had two muscle biopsies and 1 patient three muscle biopsies available in the Myology Institute Lab). Age at muscle biopsy ranged from 1 day of life (30 weeks of adjusted gestational age) to 76 years (median 16 years, IQR 3-34). Open muscle biopsies were obtained from deltoid or quadriceps muscles in most of patients. Histological and histochemical slides were systematically re-analysed by two authors (MG and NBR) with experience in skeletal muscle morphology, blinded to clinical and molecular data. For the oldest, deteriorated or not interpretable slides, new slides were obtained from the best muscle specimen available in the lab. Conventional histological and histochemical techniques, 8-10 μm thick cryostat sections were stained with haematoxylin and eosin (HE), modified Gomori trichrome (GT), Periodic acid Schiff technique (PAS), Oil red O, reduced nicotinamide adenine dinucleotide dehydrogenase-tetrazolium reductase (NADH-TR), succinic dehydrogenase (SDH), cytochrome c oxidase (COX), and adenosine triphosphatase (ATPase) preincubated at pH 9.4, 4.63, 4.35. Digital photographs of biopsies were obtained with a Zeiss AxioCam HRc linked to a Zeiss Axioplan Bright Field Microscope and processed with the Axio Vision 4.4 software (Zeiss, Germany). Fibre type pattern was determined in ATPases reactions, and by calculating the percentage of type 1 and type 2 fibres. We considered type 1 fibres predominance when there were more than of 60% type 1 fibre in deltoid muscles, and more than 40% in quadriceps muscle. CFTD was considered when all the type1 fibres were consistently (at least 35-40%) smaller than type2 fibres in absence of other pathological findings. Centronuclear pattern was considered only when myonuclei were centrally placed in almost 50% of muscle fibres showing nuclear internalization. Rods were considered in the presence of numerous, multiple, small nemaline bodies both in cytoplasmic or subsarcolemmal areas. Central cores were considered when single or multiple sharply demarked ovoidal areas devoid of oxidative stains were observed in transversal sections of type1 muscle fibres, centrally or peripherally placed. Multiminicore were considered in the presence of boundless, small areas of decreased enzymatic activity at oxidative stains [30]. “Dusty cores” were defined as irregular areas of reddish-purple granular material deposition at GT stain corresponding to decreased or/and increased enzymatic activity at oxidative stains and devoid of ATPase activity. Patients with available ultrastructural study were finally classified considering both histological and ultrastructural features. In the five patients with two or three muscle biopsies, final morphological classification was reached considering both muscle biopsies and most relevant findings.

### Immunohistochemical (IHC) study

IHC was performed in new sections from available frozen muscle samples of enrolled patients, rejecting oldest and/or deteriorated specimens. Finally, IHC analysis was available for 23 muscle biopsies. Antibodies against Desmin (Anti-Human Desmin, Clone D33, Dako Laboratories, Denmark A/S ), Myotilin (NCL-Myotilin, Novocastra Laboratories, Newcastle Upon Tyne, United Kingdom) and αB-crystallin (CRYAB, GeneTex International Corporation, Irvine, USA) were visualized using immunoperoxidase techniques. Immunofluorescence study was performed for RyR (anti-Ryanodine Receptor, clone 34C, Sigma Laboratories, Saint Louis, Missouri, USA), DHPR (anti-CACNA1S, ab2862, abcam Laboratories, Cambridge, UK) and alpha-actinin (anti-α actinin sarcomeric, clone EA-53, Sigma Aldrich Laboratories, Saint Louis, USA) antibodies on 10-μm-thick cryosections over night at 4°C. Subsequently, sections were incubated with appropriate conjugated secondary antibodies (Alexa Fluor-488 goat anti-rabbit antibody and Jackson IR goat anti-mouse antibody) for one hour. A set of control slides was prepared with omission of the primary antibodies.

### Electron microscopy (EM) study

Ultrastructural study was newly performed in all available muscle biopsies. EM images were obtained for 39 muscle biopsies. Small muscle specimens were fixed with glutaraldehyde (2.5%, pH 7.4), post fixed with osmium tetroxide (2%), dehydrated and embedded in resin. Longitudinally oriented ultra-thin sections were obtained at different level of deepness from 1 to 3 small blocks and stained with uranyl acetate and lead citrate. Ultra-thin sections of transversally oriented blocks were obtained only for the most significant findings. The grids were observed using a Philips CM120 electron microscope (80 kV; Philips Electronics NV, Eindhoven, The Netherlands) and were photo documented using a Morada camera (Soft Imaging System).

### Western blot (WB) analysis

The amount of RyR1 in muscle samples was determined by quantitative Western Blot (WB) analysis using antibodies directed against RyR1 [24] and normalized to the amount of myosin heavy chain as described previously [26]. Briefly, the muscle sample (20-40mg) was homogenized in 200 mM sucrose, 20mM HEPES (pH 7.4), 0.4mMCaCl2, 200mM phenylmethylsulfonyl fluoride, 1 mM diisopropyl Fluorophosphate using a Minilys homogenizer (Bertin, France). After electrophoretic separation on a 4–20% gradient acrylamide gel (Biorad, France) and electrotransfer to Immobilon P (Biorad, France) during 4h at 0.8A to ensure a complete transfer of the loaded proteins, the membrane was incubated with anti-RyR1 antibodies and then HRP-labelled secondary antibodies (Jackson ImmunoResearch Laboratories). Signal quantification was performed using a ChemiDoc Touch apparatus (Biorad, France) and the Image Lab software (Biorad). The total amount of RyR1 in each experiment was corrected from the amount of myosin and normalized to the amount of RyR1 present in the control referred as 100% as described previously [6]. Controls (muscle biopsy from individuals non-affected by neuromuscular disease) of different age have been used: 10 days, 3 years, 23 years and 46 years, and the amount of RyR1 in the muscle biopsy of the patient was compared to the age-related control. For each muscle biopsy, at least 3 western blots have been performed, and the value for each patient is presented as mean ± SEM of the different western blots.

### Statistical analysis

Data have been expressed as range, median and interquartile range (IQR) for continuous variables (age and % of RyR expression) and as absolute values and frequencies for categorical variables (gender, disease severity, age at onset, morphology and occurrence of ocular involvement). Age at disease onset has been categorized as <1 year or ≥1 year, based on the median value in the sample. Chi-Square test has been used to compare categorical variables, while Kruskal-Wallis or Mann-Whitney tests have been used to evaluate the distribution of continuous variables with respect to demographic, clinical and morphological characteristics. P values lower than 0.05 were considered to be statistically significant. Statistical Package for Social Science (SPSS®) version 20.0 (IBM Corp. Released 2011. IBM SPSS Statistics for Windows, Version 20.0. Armonk, NY: IBM Corp.) was used for statistical analysis.

## Results

Overall morphological, clinical and genetic data are summarized in Table 1.

**Table 1:** Morphological, clinical and genetic data overview

Patient	Revised Morphology	Clinical Severity	Allele 1	Allele 2	RYR1 (WB)	Ref.
p1	DuCD	severe	c.3223C>T	c.7025A>G	39%	this report
p2	DuCD	moderate	c.8692+131G>A	c.8692+131G>A	25%	[4]
p3	DuCD	moderate	c.9413C>T	c.11314C>T	34%	this report
p4	DuCD	severe	c.9758T>C	c.8953C>T	n.a.	[6]
p5	DuCD	severe	c.14170A>C	c.13949T>C	n.a.	this report
p6	DuCD	mild	c.14731G>A	c.7006C>T	25%	this report
p7	DuCD	mild	c.14731G>A	c.10616G>A	56%	this report
p8	DuCD	mild	c.631+1G>A	c.14717C>T	n.a.	this report
p9	DuCD	severe	c.13660- ?_14646- ?del987nt.	c.10348-6C>G; c.14524G>A	7%	[1]
p10	DuCD	moderate	c.10561G>T	c.9605C>T	n.a.	[4]
p11	DuCD	mild	c.325C>T	c.6891+1G>T	n.a.	[1]
p12	DuCD	severe	c.14524G>A; c.10348-6C>G	c.8342_8343delTA	18%	[4]
p13	DuCD	moderate	c.11999_12001del	c.6933delC	25%	this report
p14	DuCD	moderate	c.6418C>T	c.14483T>G	37%	this report
p15	DuCD	moderate	c.6418C>T	c.14483T>G	32%	this report
p16	DuCD	mild	c.7372C>T	c.1897C>T	60%	this report
p17	DuCD	mild	c.2648T>C	c.9157C>T	n.a.	this report
p18	DuCD	mild	c.4711A>G;c.10097G>A;c.11798A>G	c.2361delG	33%	this report
p19	DuCD	mild	c.4225C>T	c.14126C>T	23%	this report
p20	DuCD	moderate	c.7615-3T>A	duplication at least exon 99-106	25%	this report
p21	DuCD	mild	c.4711A>G;c.10097G>A;c.11798A>G	c.14731G>A	n.a.	this report
p22	DuCD	moderate	c.14939C>T	c.12541G>A	n.a.	[4]
p23	DuCD	mild	c.9892G>A	c.4953_4970dup	18%	this report
p24	DuCD	severe	c.644G>A	c.1840C>T	n.a.	[30]
p25	DuCD	mild	c.6721C>T	c.7268T>A	42%	this report
p26	DuCD+CCD	mild	c.4711A>G;c.10097G>A;c.11798A>G	c.14537C>T	63%	this report
p27	CCD	mild	c.6617C>T	c.6617C>T	76%	this report
p28	CCD	mild	c.7304G>T	c.7304G>T	n.a.	this report
p29	CCD	mild	c.13502C>T	c.14386T>C	40%	this report
p30	CCD	mild	c.11708G>A	c.11708G>A	45%	this report
p31	CCD	severe	c.10348-6C>G; c.14524G>A	c.7324-1G>T	14%	[27]
p32	CCD	mild	c.2938C>T	c.7372C>T	47%	this report
p33	CCD	mild	c.4711A>G;c.10097G>A;c.11798A>G	c.13691G>A	n.a.	this report
p34	CCD	moderate	c.212C>A	c.6847A>C	n.a.	this report
p35	CCD	moderate	c.5036G>A	c.464A>C	51%	this report
p36	CCD	mild	c.14126C>T	c.7063C>T	52%	this report
p37	C&R	mild	c.4711A>G;c.10097G>A;c.11798A>G	c.2984G>A	60%	this report
p38	C&R	mild	c.10579C>T	c.10579C>T	n.a.	this report
p39	C&R	mild	c.14928C>G	c.4711A>G;c.9356G>A;c.10097G>A;c.11798A>G	57%	this report
p40	C&R	moderate	c.14624T>C	c.3619G>A	20%	this report
p41	C&R	mild	c.14731G>A	c.6359T>C	52%	this report
p42	C&R	mild	c.11557G>A	c.115G>A	52%	this report
p43	C&R	severe	c.14928C>G	c.2044C>T	n.a.	this report
p44	T1P+	moderate	c.12536G>A	c.9605C>T	54%	this report
p45	T1P+	moderate	c.6860C>A	c.14939C>T	n.a.	[6]
p46	T1P+	mild	c.10579C>T	c.10579C>T	n.a.	[8]
p47	T1P+	moderate	c.10579C>T	c.10579C>T	n.a.	this report
p48	T1P+	moderate	c.12861_12869dupCACGGCGGC	c.12861_12869dupCACGGCGGC	79%	this report

*DuCD* Dusty core disease, *CCD* Central core disease, *C&R* Core-rod myopathy, *T1P+* Type1 predominance “plus”, *n.a.* Not assessed

### Clinical features

Age at onset ranged from antenatal period to 66 years (median 1 year, IQR 0-7). The first symptom/sign occurred within the first year of life in 23 patients (48%), of whom five had also antenatal manifestation. Disease onset occurred after the first year of life in 25 patients (52%) of whom 4 had a relative late onset (>20 years). Motor milestones were delayed in 25 patients (52%) and 4 out of 8 severe patients never acquired ambulation. All the patients with latest onset (>45 years) had not delayed motor milestones, as well as 9 (19%) with early-infantile onset. Ocular involvement was assessed in 38 of 48 patients and showed isolated eyelid ptosis in 4 patients, isolated ophthalmoplegia (sometimes isolated upper gaze movements) in 7 and both in 9. Overall, ocular involvement occurred in 20/38 patients and prevailed in subjects with moderate/severe disease (88%) compared to those with mild disease (24%) (*p*<0.0001). Overall clinical severity was mild in 25 (52%), moderate in 15 (31%) and severe in 8 patients (17%). All the clinical data are summarised in Table 2.

**Table 2:** Clinical features

Patient	Family	Age onset	Sex	Age at last examination	Perinatal problems	Delayed motor milestones	Ocular involvement	Muscle weakness	Other clinical features	Respiratory involvement	Spine deformities	Contractures	Dysmorphism
p1	f1	B	m	45 ds	H; FP; RI	NW	Pt + Oph	G++,	-	+++	-		-
p2	f2	2 ys	m	30 ys	n.r.	yes	Oph	G, F, SW	-	++	-	CFee	LNF; HAP
p3	f3	B	m	21 ys	RD	no	Pt + Oph	d>p LL, d/p UL	-	-	-	AcR	LNF; PrG
p4	f4	B	f	12 ws	H; FP; RI	yes	no	G, F,	-	+++	Sc	H; Kn	-
p5	f5	B	f	1 ys	H; FP; RI, Art	yes	Pt	F, pUL/LL,	-	++	-	-	RtG
p6	f6	B	f	16 ys	FP	yes	no	F, A, pUL/LL, dUL,	Dph	-	-	-	LNF; HAP;
p7	f7	60 ys	m	76 ys	no	no	Oph	F, A, pUL, dUL>LL,	Dph	-	-	-	-
p8	f8	2 ys	f	27 ys	no	yes	no	G, F	EI	+	Sc, Ld	-	HAP, RtG,
p9	f9	A	m	7 ys	O	yes	Pt + Oph	F, A, pUL/LL	Dph, CH	-	-	pUL HypL	PctE
p10	f10	B	f	34y	n.r.	n.r.	Oph (UG only)	G, F,	SW	++	Sc; Ld; SpB	AcR; H; Kn HypL;	-
p11	f11	B	m	48y	FP	n.r.	Pt + Oph	G, F,	-	+	Sc	-	LNF; HAP
p12	f12	B	f	4 ys	H; RI	yes	Pt + Oph	G, F,	PP	++	yes?	AcR; H, Kn;	HAP
p13	f13	B	f	29 ys	H	n.r.	Pt + Oph	G, F,	Dph	-	-	-	LNF
p14	f14	B	f	10 ys	H; FP, HD	yes	n.r.	pLL	Gowers	-	-	-	-
p15	f14	B	f	3 ys	H; FP, HD	yes	n.r.	pLL	-	-	-	-	-
p16	f15	10 ys	f	69 ys	no	no	no	p/ dLL	Gowers	-	-	AcR	-
p17	f16	6 mo	f	34 ys	no	yes	no	A, pUL,	CH	+	Ld; Sc	-	-
p18	f17	20 ys	f	26 ys	n.r.	yes	no	F, A, p/d LL	EI	-	no	-	-
p19	f18	1 ys	m	4ys	no	yes	Pt	F, A, pLL	-	-	-	-	-
p20	f19	B	m	25 ys	H;	yes	Oph	G, F	Dph	++	-	TFL; H; Kn; T-M	LNF; HAP
p21	F20	1 yr	f	5 ys	no	yes	no	F, pLL>UL	-	-	no	G HypL	No
p22	f21	A	f	25 ys	no	yes	Oph	A, pLL>UL, dUL/LL,	Dpg; Dph	+++	Ld	RSs; finger ext; Eb; AcR	LNF; HAP
p23	f22	1 ys	m	8 ys	no	yes	no	F, pUL/LL	-	-	Ld	-	-
p24	f23	A	m	6 ys	PO, FAS	NW	Pt + Oph	G, F	CD	-	-	G	-
p25	f24	1 ys	m	43 ys	no	yes	Oph (UG only)	F, pUL/LL, dUL	-	-	-	Kn	-
p26	f25	10 ys	f	32 ys	no	no	no	F, A, pUL/LL	Gowers	-	no	-	-
p27	f26	6 ys	f	16 ys	no	no	n.r.	A	-	-	Ky	-	-
p28	f27	1 ys	f	8 ys	no	no	n.r.	pLL	t-tW; Gowers	-	Ld; Sc	AcR; Pes cavus	-
p29	f28	1 ys	m	23 ys	no	yes	no	A, pLL>UL	-	-	-	AcR	-
p30	f29	1 ys	f	5 ys	no	yes	no	A, pUL/LL,	-	++	Ld	AcR	-
p31	f30	B	m	n.r.	H; FP; Art	NW	Pt + Oph	G, F,	-	+++	yes	Kn; Eb; H; distal HypL	-
p32	f31	45 ys	m	52 ys	no	no	no	pLL	CH	-	-	-	no
p33	f32	8 ys	f	49 ys	no	yes	no	F, A, pLL/UL	-	-	Sc	-	no
p34	f33	B	m	5 ys	HD	yes	n.r.	pLL	Gowers	-	Ld	AcR	-
p35	f34	8 ys	m	37 ys	no	n.r.	no	A, pUL/LL, dLL,	Dph, t-tW	+	Sc	-	-
p36	f35	66 ys	f	76 ys	no	no	no	A, pLL	-	-	-	-	no
p37	f36	10 ys	f	62 ys	no	n.r.	n.r.	pLL/UL, dUL	-	-	-	-	-
p38	f37	5 ys	f	21 ys	no	no	no	F, pUL/LL	-	-	-	-	-
p39	f38	8 ys	f	63 ys	no	no	no	A, pLL>UL, dUL,	-	-	-	-	-
p40	f39	B	f	26 ys	H; FP, HD, CFee	yes	n.r.	A, pUL/LL,	-	+	Sc	AcR	-
p41	f40	8 ys	m	33 ys	no	n.r.	n.r.	pUL/LL	SW	-	-	AcR	-
p42	f41	1 ys	m	13 ys	n.r.	n.r.	no	A, pLL	-	-	Ld	-	-
p43	f42	A	f	1 d	Art, (death 1 day)	NW	n.r.	G,	LH	+++	-	Art	-
p44	f43	B	m	3 ys	FP	no	Pt	G	Gowers	-	-	distal and Eb HypL	HAP
p45	f44	1 ys	f	15 ys	Art	yes	Pt + Oph	A, pUL/LL	CH	-	Ld; Sc	-	-
p46	F37	B	f	42 ys	H	yes	Oph	G, F,	-	+	-	T-M; distal HypL	HAP
p47	F37	3 ys	f	4 ys	no	no	n.r.	pUL/LL	-	-	-	-	-
p48	f45	B	m	19 ys	H	yes	Pt	G, F,	HL	+++	Sc	CFee	-

*B* At birth, *A* Antenatal, *H* Hypotonia, *RI* Respiratory insufficiency, *FP* Feeding problems, *O* Oligohydramnios, *PO* Polyhydramnios, *FAS* Fetal akinesia syndrome, *NW* Never walk, *Art* Arthrogryposis, *HD* Hip dysplasia, *Pt* Eyelid ptosis, *Oph* Ophthalmoplegia, *UG* Upper gaze, *G* Generalized, *SW* Scapular winging, *d* Distal, *p* Proximal, *UL* Upper limbs, *LL* Lower limbs, *F* Facial, *Ax* Axial, *Dph* Dysphonia, *Dpg* Dysphagia, *EI* Exercise intolerance, *CH* Calf hypertrophy, *PP* Precocious puberty, *CD* Cognitive delay, *t-tW* Tip-toe walking, *HL* Hearing loss, *LH* Lung hypoplasia, *Sc* Scoliosis, *Ld* Lordosis, *Ky* Kyphosis, *SpB* Sspina bifida, *AcR* Achilles tendon retraction, *TFL* Tensor fascia lata, *T-M* Temporo-mandibular, *RSs* Rigid spine, *CFee* Club feet, *LNF* Long narrow face, *HAP* High arched palate, *PrG* Prognathism, *RtG* Retrognathia, *PctE* Pectus excavatum, *HypL* Hyperlaxity, *Kn* Knee, *Eb* Elbow, *n.r.* Not reported

#### Morphological findings

**Fig. 1:**
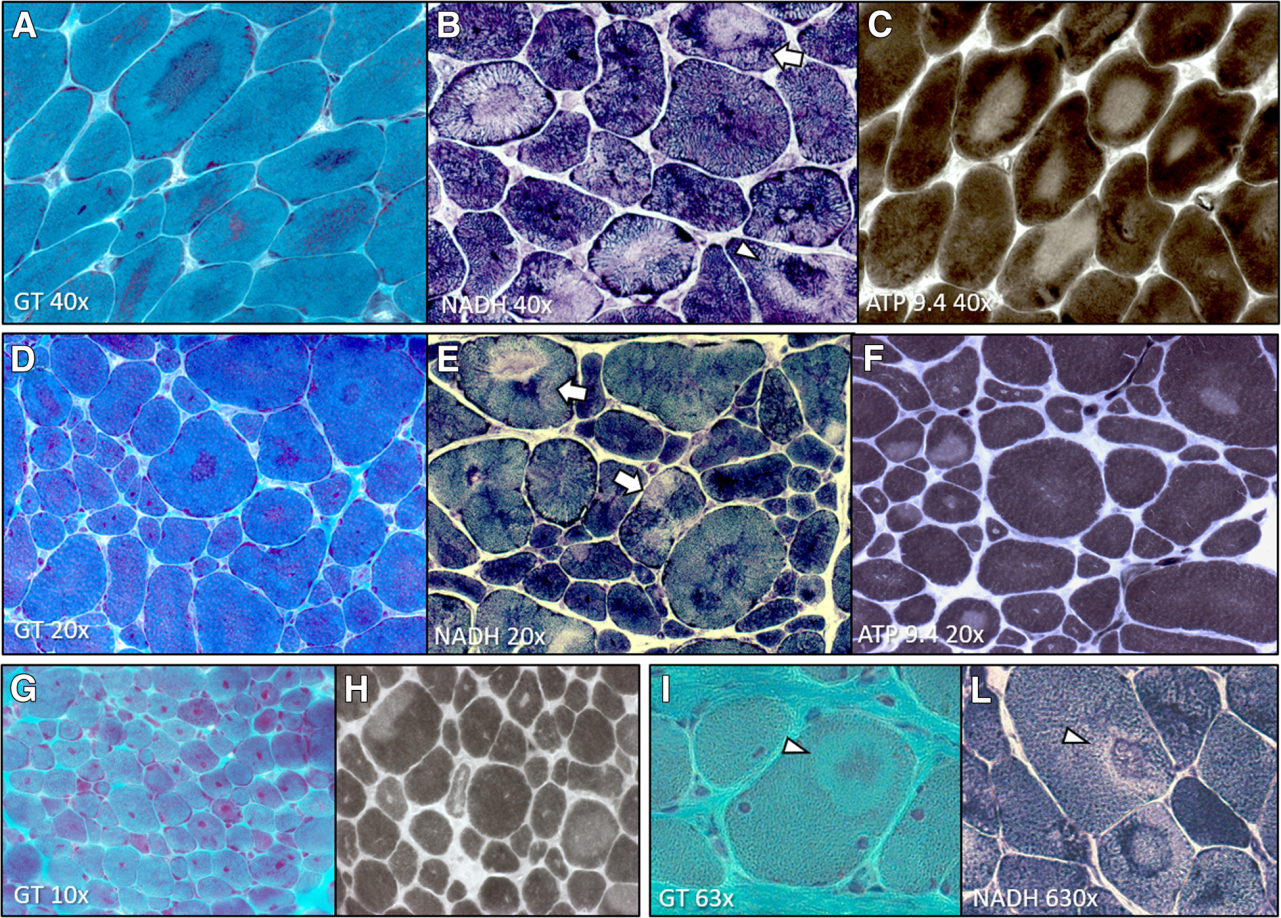
DuCD morphological spectrum. Dusty cores: irregular areas of reddish-purple granular material deposition at GT stain (**a**, **d**, **g** and **i**) corresponding to areas of devoid ATPase activity (**c**, **f** and **h**) and decreased or increased enzymatic activity at oxidative stains (**b**, **e** and **l**), sometime occurring in the same core side-by-side (**b** and **e**; arrows) or concentric with “targetoid” appearance (**b**, **i** and **l**; arrowheads). Note type 1 fibre uniformity (**c**, **f** and **h**) and prominent nuclear internalization (**d** and **g**). (**a**-**c**: p12, 28 years; **d**-**f**: p11, 48 years; **g** and **h**: p19, 4 years; **i** and **l**: p24, 1 year)

We defined “dusty core fibres” the muscle fibres with irregular areas of reddish-purple granular material deposition at GT stain corresponding to large areas of uneven myofibrillar disorganisation at oxidative stains, characterised by blended decreased or/and increased enzymatic activity and devoid ATPase activity (“dusty cores”) (Fig. 1a-f). These dusty core fibres are different from the classic central cores observed in CCD because the last cores are well delimited and have not fuchsine deposit inside the core.

Dusty cores were the most frequent histopathological lesion, detected in 32 muscle biopsies and confirmed by EM in 30 muscle biopsies (56,6%). We named “Dusty Core Disease” (DuCD) the corresponding group of congenital core myopathy with dusty cores.

Central cores (single or multiple, centrally or peripherally placed) were observed in 10 (18,5%) muscle biopsies, whereas core-rod association was detected in 7 (13%) biopsies. Type1 fibre predominance/uniformity was the main histopathological alteration in 5 (9,2%) muscle biopsies, associated to minimal changes of the myofibrillar network at oxidative stains (type1 predominance “plus”, T1P+). Finally, 2 muscle biopsies were classified as CNM with type1 fibre hypotrophy, both performed at early stages of life.

The number of muscle fibres containing dusty cores ranged greatly from few fibres up to 30-40% of fibres in muscle biopsy.

Dusty cores were consistently associated to type1 fibre predominance or uniformity and prominent nuclear internalization and centralization. Some muscle biopsies presented some supplementary peculiar features: in 3 muscle biopsies (p4, p14, p17) dusty cores were detected only in few (2-5) muscle fibres only after an extensive and careful revision; whereas in 3 others (p14, p15, p21) none or minor nuclear internalization was observed; 3 muscle biopsies (p9, p18, p24) presented a high fibre size variability with hypertrophic and atrophic fibres, fibre slitting, increased connective tissue without necrosis and regeneration (pseudo-dystrophic appearance); five muscle biopsies (p1, p11, p16, p19, p20) showed a prominent nuclear internalization and centralization (CNM-like) (Fig. 1g,h); one case (p26) showed surprisingly both dusty and central cores. These findings provide the evidence that a certain variability exists among the DuCD morphological spectrum, in which dusty cores represent the unifying lesion.Regardless of these peculiarities, in 14/31 cases (45%) dusty cores presented a “targetoid” appearance in variable amount of muscle fibres (Fig. 1i,l).

**Fig. 2:**
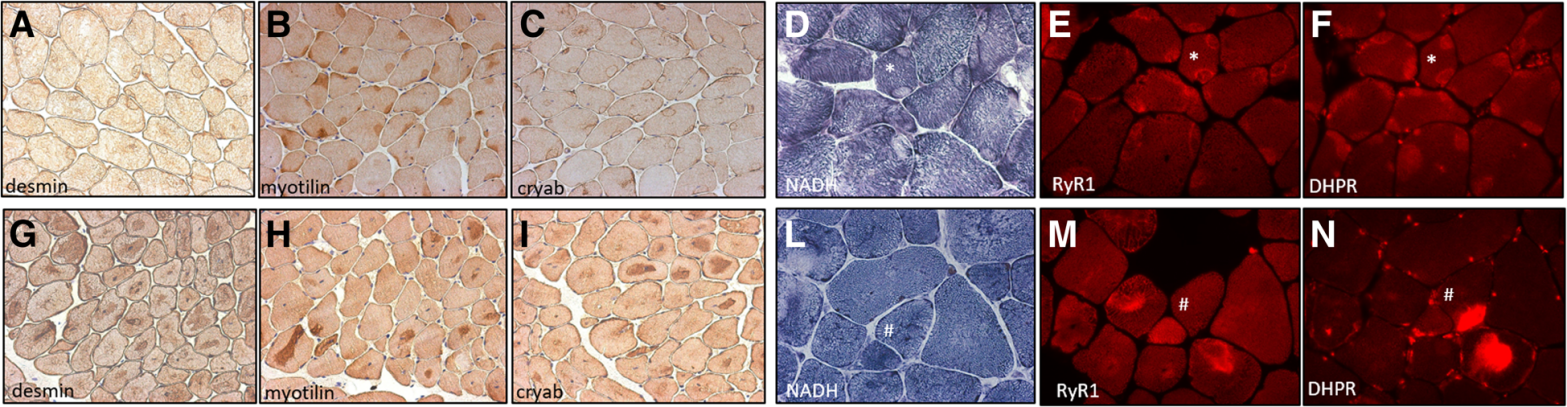
Immunoistochemestry and Immunofluorescence of DuCD and CCD. IHC showing positive immunostaining for desmin (**a** and **g**) myotilin (**b** and **h**) and αB-cristallim (**c** and **i**) in both central cores (**a**-**c**) and dusty cores (**g**-**i**). Serial images of NADH and immunofluorescence for RyR1 and DHPR showing positive signal in central cores (**e** and **f**) and positive, but unmatching signal in dusty cores (**m** and **n**). (**a**-**c**: p26, 32 years; **d**-**f**: p40, 25 years; **g**-**i**: p13, 28 years; **l**-**n**: p7, 76 year)

The IHC study for myofibrillar proteins (desmin, myotilin, αB-crystallin) did not show any specificity in type1 predominance group. By contrast central core and dusty core groups showed an inconsistently positive immunostaining of all three antibodies inside cores (Fig. 2a-c, g-i), with the only peculiarity for desmin which showed a peripheral halo of cores in some biopsies of CCD group. Likewise, immunofluorescence for RyR1 and DHPR showed an inconstant positivity for central core and dusty core groups, even if in DuCD group positive signal by RyR1 and DHPR did not match (Fig. 2d-f, l-n). No specificity was observed for type1 predominance group and by a-actinin immunostaining for all groups.

**Fig. 3:**
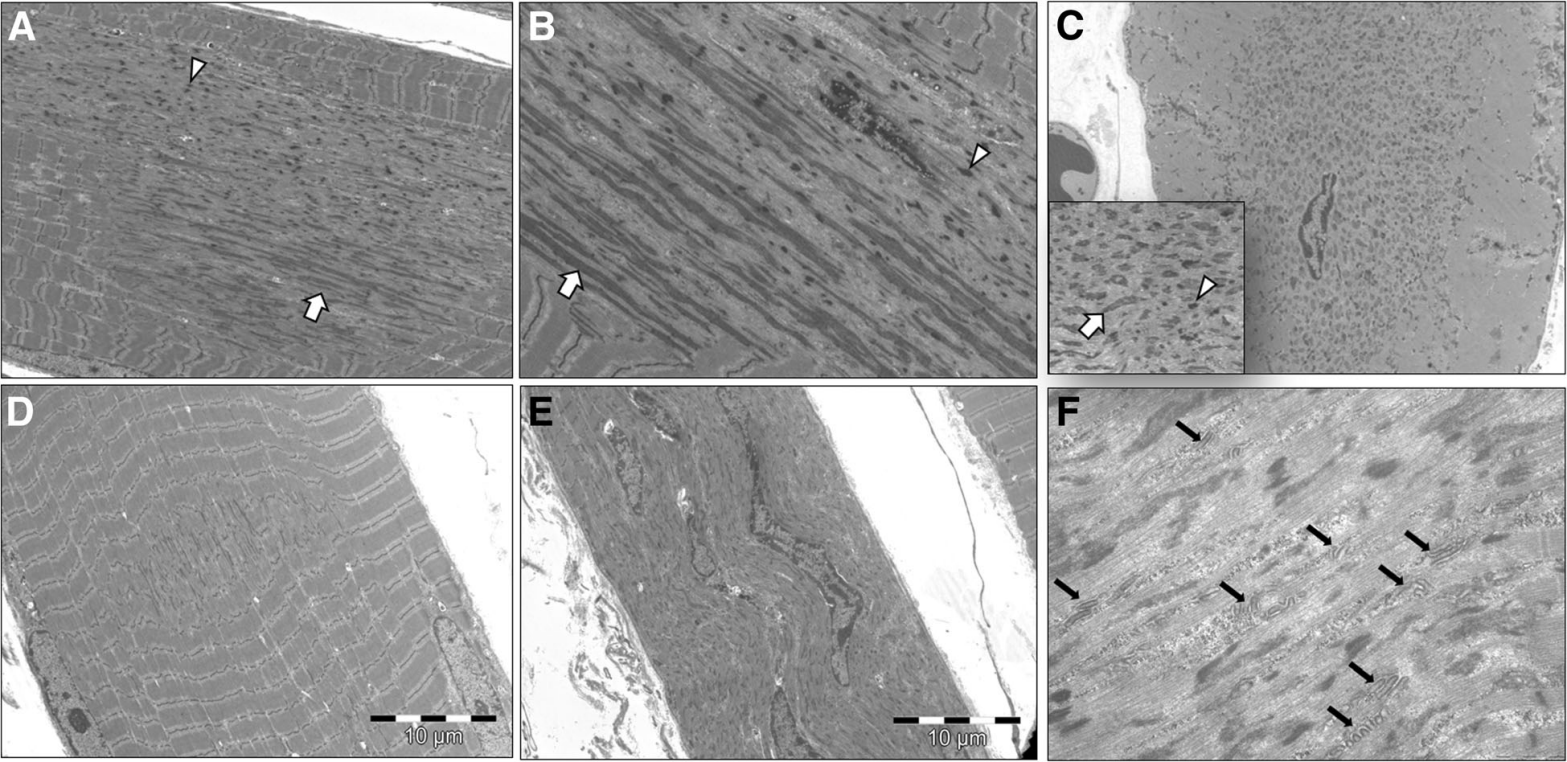
Ultrastructural findings of DuCD. Large irregular areas of sarcomeric disorganization with longer longitudinal axis and shorter transversal axis (**a**) containing abundant electrodense longitudinally-smeared material (arrows) and thickened short Z-line darker fragments (arrowheads), in longitudinal (**a** and **b**) and trasversal (**c**) sections. Smaller areas of sarcomeric disorganization (few sarcomeres) were alsodetected (**d**). Areas of disorganization occupying the entire muscle fibre in width with centralized/internalised nuclei (**e**). Several triads duplication or multiplication were detected inside dusty cores (black arrows). (**a**, **d** and **e**: p10, 34 years; **b**: p17, 34 years; **c** and **f**: p7, 76 year)

The EM study showed some peculiarities in the DuCD group. In longitudinal sections, the areas of sarcomeric disorganization consisted of cellular debris with sarcomeric fragments of thin and thick filaments devoid of mitochondria. In addition, two abnormal elements were consistently observed: abundant electrodense longitudinally-smeared material and thickened short Z-line darker fragments, sometime superposed (Fig. 3a,b). These features were evident also in transversal sections (Fig. 3c), inside areas of disorganization which frequently showed sub-areas of electrodense material deposition, mixed close sub-areas of less osmophilic material. In longitudinal sections, areas of sarcomeric disorganization ranged from few to more than 50 sarcomeres (Fig. 3a). In all cases of dusty core group, smaller areas of sarcomeric disorganization, resembling minicores, were noticed (Fig. 3d). The larger areas of disorganization, possibly corresponding to dusty cores at optic microscopy, had an asymmetric or «star-like» shape, with a longer longitudinal axis and a shorter transversal axis. Sometimes, these areas of disorganization occupy the entire muscle fibre in width (Fig. 3e). Interestingly in almost half of cases, several triads duplication or multiplication were detected inside areas of disorganization (Fig. 3f). Nevertheless, in all suspected minicore lesions by EM, a deeper analysis, performed with numerous serial sections, revealed the presence of bigger areas of disorganization. Finally, Z-line abnormalities were detected in the majority of cases, including duplication, streaming and smearing across 2-4 sarcomeres.

Both central core and core-rod groups showed at EM classic unstructured and, less frequently, structured cores. Nemaline rods were consistently observed in all cases of the core-rod group, in clusters of subsarcolemmal areas or scattered in sarcoplasm, frequently in perinuclear areas.

Overall, the ultrastructural study confirmed the optic microscopy classification in 37 out of 39 muscle biopsies analysed. All cases of core-rod were confirmed by EM. Surprisingly in one case of CCD and one case of DuCD no abnormalities were found at the EM study in the specimens analysed.

Concerning patients with more than one muscle biopsy available, the average time between the two biopsies was 12,6 years (range 6-22 years). The first muscle biopsy showed the presence of dusty cores in 3 out of 5 patients, confirmed also in the second muscle biopsy, two of which also showed a “targetoid” appearance and one a pseudo-dystrophic pattern. Interestingly, the first muscle biopsy in 2 cases showed only nuclear centralization in small type1 fibres without any myofibrillar disorganization detected by optic microscopy. Both cases revealed dusty cores in the subsequent muscle biopsies performed some years later.

Finally, based on both the histological and the ultrastructural analysis of all muscle biopsies, the 48 *RYR1*-recessive patients were classified as follow: 26 (54%) patients with Dusty Core Disease (DuCD); 10 (21%) patients with Central Core Disease (CCD); 8 (15%) patients with Cores and Rods (C&R) and 5 (10%) with Type1 predominance “plus” (T1P+). All morphological data are summarised in Table 3.

**Table 3:** Morphological data

Pt	Morph	First morph classification	Age at biopsy	muscle	Optic microscopy							Electron microscopy		
					fibre size variability	connective tissue	Nuclei	fibre type	cores	rods	other findings	cores’ characteristics	length (sarcomeres)	other relevant findings
p1	DuCD	CNM+	45 ds	n.r.	+	+	I & C++	T1P and A	dusty	no	V, CB	◊-DC containing OFSM and STDF	up to 30	SAD (>5 sarcomeres)
p2	DuCD	CNM+	26 ys	D	+	-	I & C (rare)	T1U	dusty	no	-	◊-DC containing OFSM and STDF	up to 30	TR (up to 4) inside dusty cores; SAD (5-8 sarcomeres); Z-line S+J; occasional aligned thickened Z-line fragments inside small areas of disorganization
p3	DuCD	CNM+	21 ys	D	+	-	I & C	T1P and A	dusty + targetoid	no	-	◊-DC containing OFSM and STDF	up to 20	ZB; AV; Hc; SAD (2-4 sarcomeres); aligned thickened Z-line fragments inside small areas of disorganization in 1 fibre
p4	DuCD	CNM+	20 days	n.r.	+	+	I & C	T1P (mild)	dusty (rare)	no	-	◊-DC containing OFSM and STDF; multiple	up to 30	TR inside dusty cores; SAD (2-4 sarcomeres); Z-line S (2 sarcomeres)
p5	DuCD	CCD	8 ws	n.r.	+	-	I	T1U	dusty	no	V	n.a.	-	-
p6	DuCD	CNM+	16 ys	D	+++	-	I & C	T1P and A	dusty + targetoid	no	-	◊-DC containing OFSM and STDF	up to 30	TR (up to 7) inside dusty cores; SAD (3-6 sarcomeres); mitochondrial electrodense inclusion
p7	DuCD	CCD/MFM	76 ys	D	+	-	I & C	T1P	dusty + targetoid	no	-	◊-DC containing OFSM and STDF	>20	TR inside dusty cores; perinuclear lipofuscin; SAD (3-6 sarcomeres)
p8	DuCD	CCD	23 ys	Q	++	-	I & C	T1P	dusty	no	CB	n.a.	-	-
p9	DuCD	CCD	1 ys	Q	+++	++	I	T1P	dusty	no	-	◊-DC containing OFSM and STDF	>15	TR inside dusty cores; SAD (5-6 sarcomeres); Z-line S
p10	DuCD	CMN+/MmD	34 ys	D	+		I & C	T1P	dusty	no	-	◊-DC containing OFSM and STDF	up to-30	TR inside dusty cores; SAD (4-5 sarcomeres)
p11	DuCD	CNM/CCD	48 ys	n.r.	+	+	I & C++	T1U	dusty + targetoid	no	-	◊-DC containing OFSM and STDF	>30	CNM-like features with perinuclear disorganization and vacuolization and nuclear chains; occasional multiple areas of disorganization in the same fibre; SAD (few sarcomeres)
p12	DuCD	CNM	15 days	n.r.	+++	-	I & C	normal	no	no	-	n.a.	-	-
		CNM+	4 mos	D	+++	+	I & C	T1P (mild)	dusty	no	V	◊-DC containing OFSM and STDF	>15	-
		CNM+	12 ys	P	+	+/-	I & C	T1U	dusty + targetoid	no	-	◊-DC containing OFSM and STDF	>20	-
p13	DuCD	CNM	6 ys	n.r.	+	-	I & C	normal	no	no	-	n.a.	-	-
		CNM+	28 ys	D	+	+	I & C	T1U	dusty + targetoid	no	-	◊-DC containing OFSM and STDF	>20	TR inside dusty cores; inverted triads (T-tub/SR/T-tub); abundant T-tubules; SAD (4 sarcomeres); Z-line J
p14	DuCD	CCD	9 ys	D	+	+	normal	T1P	dusty (rare) + targetoid	no	-	◊-DC containing OFSM and STDF	up to 30	TR (up to 5) inside dusty cores; occasional aligned thickened Z-line fragments inside small areas of disorganization
p15	DuCD	CCD	3 ys	n.r.	+++	++	normal	T1P	dusty	no	-	n.a.	-	-
p16	DuCD	CNM/MmD	57 ys	D	+	-	I & C++	T1P	dusty + targetoid	no	-	◊-DC containing OFSM and STDF	up to 30	Z-line S+J; inside small areas of disorganization; SAD (2-4 sarcomeres)
p17	DuCD	CCD/MmD	34 ys	D	+	-	I & C	T1P	dusty (rare)	no	-	◊-DC containing OFSM and STDF	up to 40	TR inside dusty cores; aligned thickened Z-line fragments in the peripheral areas of sarcomeric disorganization; SAD (2-3 sarcomeres); Z-line S
p18	DuCD	CCD/C&R	25 ys	Q	++	++	I	T1U	dusty + targetoid	no	lipid	◊-DC containing OFSM and STDF, targetoid	only transversal	TR + inverted duplicated triads (T-tub/SR/T-tub)
p19	DuCD	CNM+	4 ys	Q	+	-	I & C++	normal	dusty	no	-	◊-DC containing OFSM and STDF	up to 15	TR (up to 6) inside dusty cores; SAD (5 sarcomeres)
p20	DuCD	CNM/NM	7 ys	TFL	+	+	I & C++	T1P	dusty	no	V, CB	◊-DC containing OFSM and STDF	up to 30	TR inside dusty cores; SAD (3-4 sarcomeres)
		NM/vacuolar	15 ys	P	+++	+/-	I & C	T1P	dusty	no	V	n.a.	-	-
p21	DuCD	CNM+	5 ys	Q	+	++	I (mild)	T1U	dusty + targetoid	no	-	n.a.	-	-
p22	DuCD	CNM+	21 days	D	+	-	I & C	T1P & A	dusty	no	-	◊-DC containing OFSM and STDF	up to 15	SAD (2 sarcomeres)
		CNM+	12 ys	D	+	+	I & C	T1U	dusty + targetoid	no	-	normal	-	-
p23	DuCD	CNM+	8 ys	Q	+	+	I	T1U	dusty + targetoid	no	-	◊-DC containing OFSM and STDF	>20	SAD (6 sarcomeres)
p24	DuCD	CCD	15 days	Q	+++	++	I	T1P	dusty	no	-	n.a.	-	-
		CCD	1 ys	D	+++	++	I & C	T1P	dusty + targetoid	no	V		-	-
p25	DuCD	CNM+	43 ys	D	+	+	I & C	T1P	dusty + targetoid	no	-	◊-DC containing OFSM and STDF	up to 20	TR inside dusty cores; AV; medium-size (9-10 sarcomeres) areas of disorganization
p26	DuCD+CCD	CCD	32 ys	D	+/-	+	I	T1P	eccentric + dusty (rare)	no	-	◊-DC containing OFSM and STDF + UC	up to 20	sarcomeric duplication (titin-like); SAD (3 sarcomeres)
p27	CCD	CCD	16 ys	D	+	-	normal	T1P	eccentric (rare)	no	-	n.a.	-	-
p28	CCD	CCD	n.r.	Q	+	-	I (mild)	T1P	eccentric	no	-	n.a.	-	-
p29	CCD	CCD	23 ys	D	+/-	-	I	T1U	multiple	no	-	SC (multiple)	>50	SAD (<10 sarcomeres); Z-line S
p30	CCD	CCD	5 YS	Q	+/-	+/-	normal	T1P	central	no	-	n.a.	-	-
p31	CCD	CCD	n.r.	n.r.	+/-	-	normal	T1P	eccentric	no	-	n.a.	-	-
p32	CCD	CCD	47 ys	n.r.	+/-	-	I	T1P	multiple eccentric	no	-	n.a.	-	-
p33	CCD	CCD	46 ys	n.r.	+	+	I	T1U	central	no	-	UC	>30	-
p34	CCD	CCD	n.r.	n.r.	+	-	I	T1P	central	no	-	n.a.	-	-
p35	CCD	CCD	35 ys	D	+	+	I	T1U	multiple	no	-	UC	>50	small to large areas of disorganization with Z line duplication
p36	CCD	CCD	76 ys	D	+	+	I	T1P	eccentric	no	-	normal	-	-
p37	C&R	CCD	62 ys	D	+	-	I	T1P	dusty-like	yes	-	UC	>15	small (<5 sarcomeres) areas of desorganization
p38	C&R	CCD	21 ys	Q	+	+	I	T1U	eccentric	yes	-	UC	>20	small (<3 sarcomeres) areas of desorganization
p39	C&R	C&R	57 ys	D	+/-	-	normal	T1P &	eccentric	yes	-	UC	>20	small (<5 sarcomeres) areas of desorganization
p40	C&R	C&R	25 ys	D	+	-	normal	T1U	multiple	yes	-	UC & SC	up to 20	small (<3 sarcomeres) areas of desorganization
p41	C&R	C&R	33 ys	D	+	+	I	T1U	eccentric	yes	-	UC & SC	up to 30	-
p42	C&R	CCD	2 ys	D	+	+	normal	T1P	eccentric	yes	-	UC & SC	>15	Focal areas of disorganization (2 sarcomeres) of z-line smearing
p43	C&R	NM/CMD	1 day	n.r.	++	++	I (mild)	T1P & A	dusty-like	yes	-	UC	-	-
p44	T1P+	T1P	3 ys	n.r.	+	+	I (mild)	T1P & A	MFD (mild)	no	-	n.a.	-	-
p45	T1P+	T1P	3 ys	n.r.	+	+	I	T1U	normal	no	-	no	-	cisternae enlargement
p46	T1P+	T1P	2 ys	D	+/-	-	normal	T1U	MFD (rare)	no	-	no	-	-
p47	T1P+	T1P	4 ys	n.r.	-	+	normal	T1U	MFD (mild)	no	-	no	-	-
p48	T1P+	T1P	14 ys	n.r.	+	-	normal	T1P	MFD (mild)	no	Alv	no	-	unstructured areas with mitochondria

*DuCD* Dusty core disease, *CCD* Central core disease, *C&R* Core-rod myopathy, *T1P+* Type1 predominance “plus”, *CNM* Centronuclear myopathy, *MmD* Multiminicore disease, *MFM* Myofibrillar myopathy, *CMD* Congenital muscular dystrophy, *NM* Nemaline myopathy, *D* Deltoid, *Q* Quadriceps, *P* Paravertebralis muscles, *TFL* Tensor fascia lata, *I* Internalised, *C* Centralised, *T1P* Type1 predominance, *T1U* Type1 uniformity, and *A* Atrophy, *◊-DC* Irregular/star-shaped dusty core, *OFSM* Osmophilic filamentous smeared material, *STDF* Short thickened darker fragments, *UC* Unstructured cores, *SC* Structured cores, *SAD* Small areas of myofibrillar disorganization, *TR* Triad replication, *ZB* Zebra bodies, *AV* Autophagic vacuoles, *Z-line S* Streaming; Z-line, *J* Jagging, *Hc* Honeycomb figures

### Molecular data

Every patient has at least one variation on each allele of *RYR1* gene (Additional file 1). Each variant was identified based on its low frequency (less than 0.5% in database gnomAD). Thirteen variants were not previously reported. Twenty-six hypomorphic variants were found (non-sense, out of frame deletion, splice variant). The others were in frame deletion (p.M4000del), in frame duplication (p.E1651_L1656dup), missense variants and a combination of 3 or 4 variants resulting in the mutant haplotype p.[I1571V;R3366H;Y3933C] or p.[I1571V; R3119H; R3366H; Y3933C] already described [10]. Each variant was found in a conserved region (in the vertebrate subphylum). Only L4650P, R2140W, L883P, P4501L and R4179H variations were classified as moderate, a similar type of amino acid change being observed in other species of vertebrate phylum. All the bioinformatic scores lead to consider all these variations as impacting the protein. Each variant was localized on the RyR1 3D structures and 26 variants were predicted to induce destabilisation by foldX software. Other 10 variants, localized on the RyR1 3D structures (in the so-called domains 5gl1 and 5taz), were predicted to induce the loss of nearby interaction. Five variants (N2342S, R4179H, V4842M, E4911K) were predicted to induce no particular change in RyR1 structure at the interaction or steric hindrance level [3, 8]. The variant V4842M was associated with an additional splice variant. The N2342S has already been associated with a functional defect, the E4911K is located in the triadin interaction motif, the A4946V is located in a transmembrane domain and the R4179H is located in a calcium affinity domain [38]. Overall, 17 different missenses variants (40.5%) are located in the bridge solenoid (B-sol) domain (which contains the FKBP binding domain), 2 in the S2S3 domain, 4 in the Csol (5%), 3 in the J sol (7.5%), 5 in the NTD domain (12.5%), 6 in the pore domain (15%), 3 in the pVSD domain (7.5%), 1 in the RYR1&2 domain (2.5%), 2 in the SPRY1 (5%), 2 in the SPRY2 (5%), 1 in the SPRY3 (2.5%), 3 in the TaF domain (7.5%) and 1 in undefined regions (2.5%). For the haplotype variant [I1571V; R3366H; Y3933C], the variants were respectively localized in SPRY3, BSol and CSol domains. Among all mutations only 2 were previously associated to MHS (T2206M, R2458C). To note 5 were located in MH1 domain (p.E39K, p.S71Y, p.Q155P, p.G215E, p.R614C), 6 in the MH2 domain (p.T2206M, p.N2283H, p.R2355W, p.M2423K, p.R2435L, p.R2458C) and 17 in the MH3 domain (p.Y3933C, p.M4000del, p.R4179H, p.E4181K, p.A4287_A4289dup, p.P4501L, p.R4564Q, p.L4650P, p.T4709M, p.K4724Q, p.Y4796H, p.L4828R, p.V4842M, p.A4846V, p.M4875T, p.A4906V, p.E4911K). Among all variants only 2 were previously associated to MHS (T2206M, R2458C). According to the ACMG classification, 19 variants were considered as class 5 (pathogenic), 31 variants were classified as class 4 and 19 variants of unknow signification (class3).

### *RYR*1 expression study

RyR1 expression was obtained from 31/54 muscle biopsies: 17 from DuCD group (68%), 7 CCD (70%), 5 C&R (62%) and 2 T1P+ (40%). All the patients had reduced expression of RyR1 in muscle tissue (median 40%, range 7-79%, IQR 25-53). Patients with a very large reduction in the amount of RyR1 (<15% of total) were severely affected. An amount as low as 20% resulted only in a moderate phenotype in at least 5 patients. The patients with latest disease onset (>50 years) had more than 50% protein. Patients with MH mutations have a lower reduction in RyR1 amount, estimated at 7 to 9% for the R2458C variant and 11,5% for the T2206M variant. The reduction related to haplotypes ranged between 17-24%. The E4911K variant leads to a RyR1 loss estimation ranging between 22 to 37.5%.

### Clinical, morphological and genetic correlation

Most of the severe patients belonged to the DuCD group (6/8 patients) compared to CCD and C&R (1 patient in each group respectively). Among T1P+ patients, none had severe clinical presentation. Mild clinical phenotype represented the most frequent clinical presentation in CCD and C&R (70% and 62,5% respectively). Surprisingly, among T1P+ group, most of patients (80%) had a moderate clinical phenotype and only 1 (20%) a mild presentation. In DuCD group, 12 patients (48%) had a mild clinical phenotype, 8 moderate (32%) and 6 severe (20%) presentation. Nevertheless, given the small sample size, differences observed in clinical severity among different morphological groups were not statistically significant. Most of patients with ocular involvement belonged to DuCD (70%) and T1P+ (20%) groups. Conversely, 56% of DuCD and, surprisingly, 80% of patients of T1P+ group had ocular involvement. Ocular involvement was significantly more frequent in moderate and severe forms (90% and 86%) compared to mild clinical phenotype (24%) (*p*<0.0001).

**Fig. 4:**
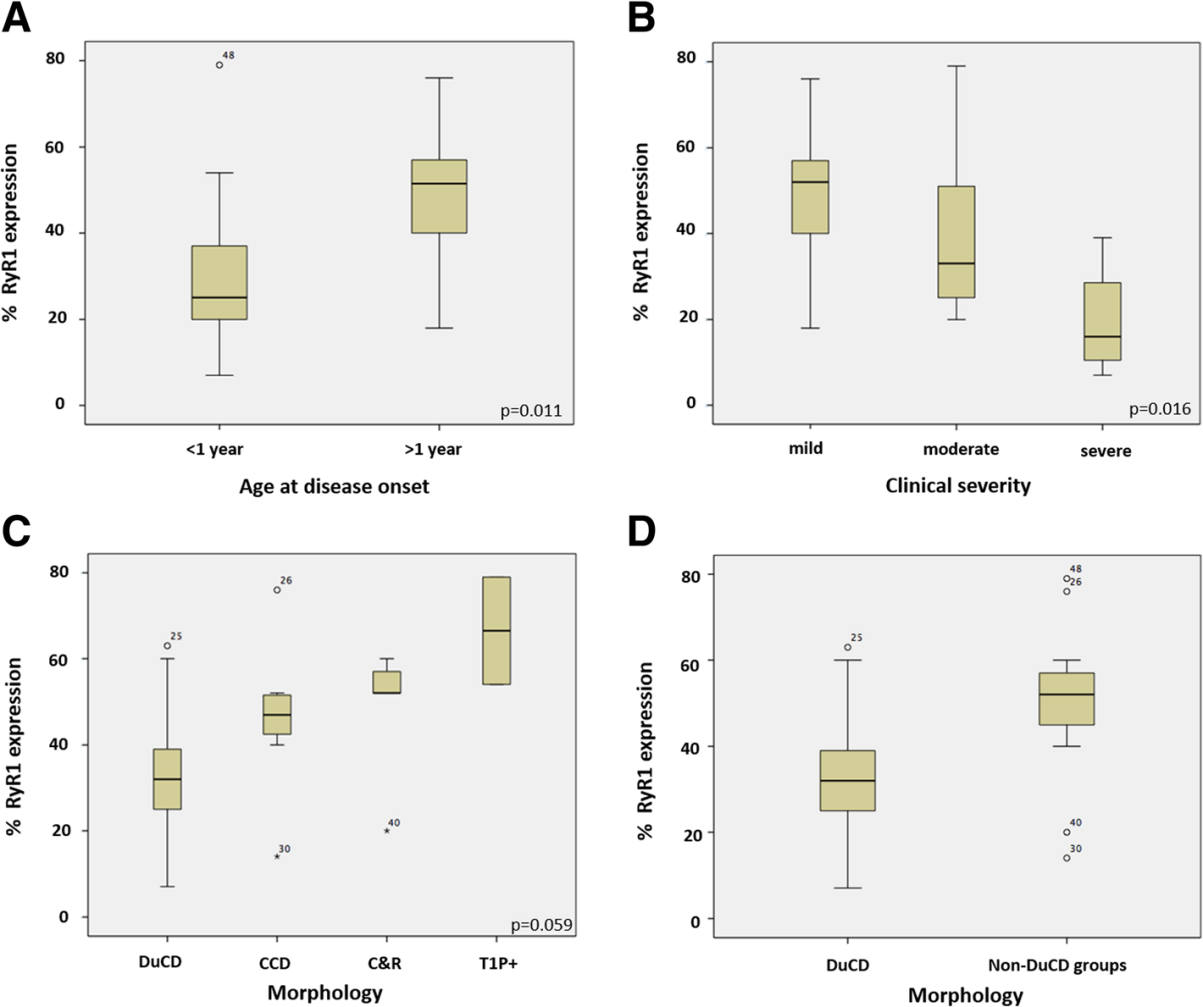
Clinical, morphological and genetic correlation. RyR1 expression was significantly lower in patients with an earlier onset (<1 year) (**a**) and more severe clinical presentation (**b**). The lowest RyR1 expression was observed in DuCD group compared to CCD, C&R and T1P+ groups alone (**c**) or together (**d**)

Percentage of RyR1 expression was significantly lower in patients with disease onset <1 year (range 7-79; median 25.00; IQR 19-38) compared to those with later onset (≥1 year) (range 18-76; median 51.50; IQR 38.25-57.75) (*p*=0.011) (Fig. 4a). An interesting correlation (Kruskal-Wallis test) was also found between clinical severity and *RYR1* expression, with lower RyR1 expression in more severe patients (p=0.016) (Fig. 4b). Concerning morphology, the lowest *RYR1* expression was observed in DuCD group (range: 7-63%; median: 32%, IQR 24.0-40.5), compared to CCD (range: 14-76%; median: 47%, IQR 40-52), C&R (range: 20-60%; median: 52%, IQR 36.0-58.5) and T1P+ (range: 54-79%) (*p*=0.059). Comparing DuCD group with non-DuCD groups, *RYR1* expression was significantly lower in the former (*p*=0.015) (Fig. 4c,d).

Concerning the position of variants across the *RYR1* gene, high prevalence (40,5%) of missense variants in BSol domain was found among the entire cohort of patients regardless of clinical and morphological phenotype. Nevertheless, no significant relationship was found between the position of variant and morphological or clinical phenotype. Interestingly, variants located in the BSol domain and the pore domain were more frequent in DuCD patients (10/19 and 9/12 respectively).

## Discussion

After a full revision of histopathological and ultrastructural features of 54 muscle biopsies we have reclassified 48 *RYR1*-recessive patients in 4 main categories: DuCD (54%), CCD (21%), C&R (15%) and T1P+ (10%). Dusty cores represent the most frequent histopathological manifestation of *RYR1*-recessive myopathies. Dusty cores represent the characteristic and unifying morphological feature in this group and are characterised by the presence of reddish-purple granular material (“dusty”) deposition at GT stain, corresponding to irregular areas of altered enzymatic activity at oxidative stains (“cores”), and devoid of ATPase activity. Unlike classic central cores, which show a sharply demarked ovoidal area devoid of oxidative stains, dusty cores have no sharply demarked borders, no round/ovoidal shape and no regular size. Moreover, reddish purple material deposition has not been reported in classical CCD due to dominant *RYR1* mutations or in *MYH7*-related core disease. In fact, no dusty cores were observed among 154 muscle biopsies of dominant *RYR1-* and 10 of *MYH7*-related myopathies examined in our lab [30, 32].

**Fig. 5:**
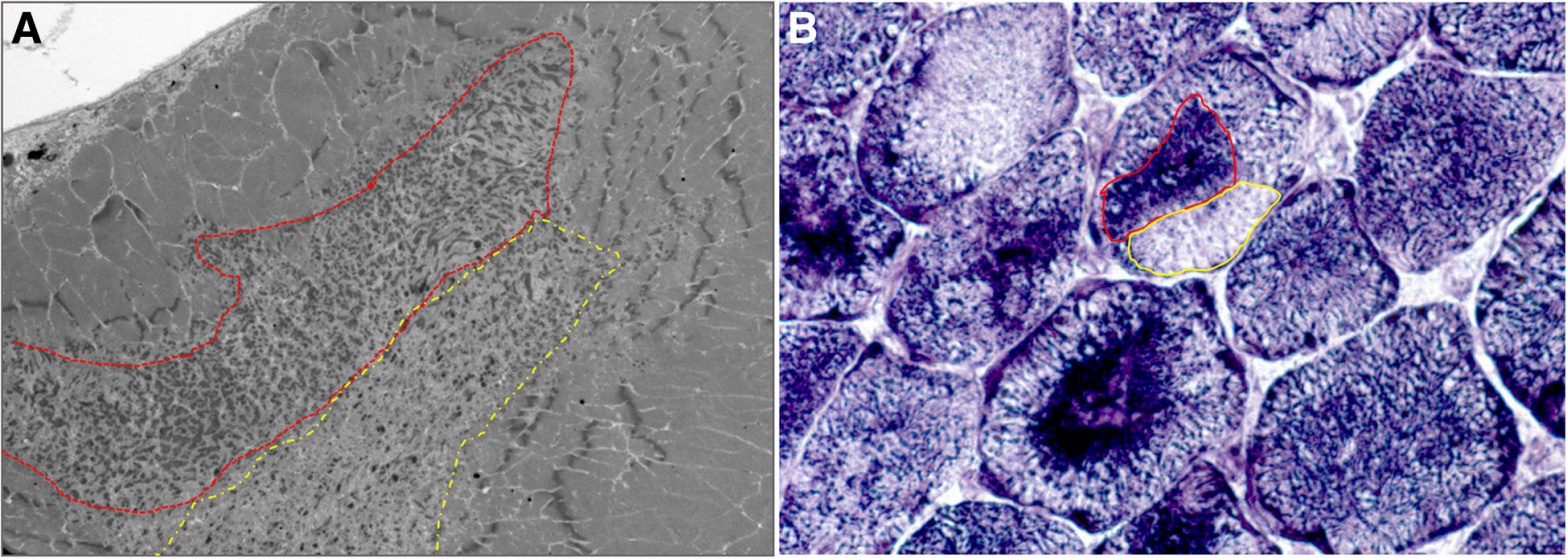
Inhomogeneous composition of dusty cores. Transversal section of dusty core by EM (**a**) showing sub-area of accumulated osmophilic material (red line) close to pale sub-area (yellow line), possibly corresponding to the peculiar aspect of increased (red line) and decreased (yellow line) enzymatic activity at NADH (**b**)

Dusty cores range from small cytoplasmic or subsarcolemmal spots to very large areas of sarcomeric disorganization, sometime occupying the entire muscle fibre in transversal sections, which is not a common finding in CCD. These findings are also detected by EM. The areas of sarcomeric disorganization are similar to the unstructured cores of CCD, but with some differences. First, dusty cores never span longitudinally the entire muscle fibres, but mostly range between 10 and 50 sarcomeres in length. Second, unlike the regular width of the core along the muscle fibre in central cores, the disorganized areas in dusty cores are irregular, enlarged in their central part, sometimes conferring an irregular longitudinal shape to the core, sometimes with “star-like” appearance. Third, composition of cores of DuCD group presents 2 main peculiarities compared to CCD: 1) in longitudinal sections, strands of longitudinally-smeared osmophilic material and darker fragments of short thickened Z-line are consistently observed and 2) in transversal sections, these elements are not homogenously distributed in the core area but are usually accumulated in certain regions, leading to sub-areas of dense material deposition close to sub-areas of less osmophilic material, probably corresponding to the peculiar aspect of mixed increased-decreased enzymatic activity at oxidative staining (Fig. 5). This phenomenon could explain the peculiar side-by-side of increased/decreased activity at oxidative stains and the unmatched immunofluorescence by RyR1 and DHPR, corresponding to the inhomogeneous composition of dusty cores. The presence of some areas of dusty cores intensely stained at NADH and devoid of mitochondria at EM, leads to speculate that material which accumulates inside dusty cores could, at least partially, belong to sarcoplasmic reticulum or other cytoplasmic structures.

**Fig. 6:**
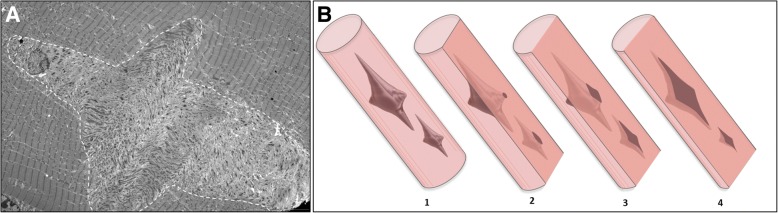
Star-shaped dusty cores and ultrastructural finding diagram. Longitudinal section of star-like shaped dusty core by EM (**a**). Three-dimensional representation of dusty cores inside muscle fibre (**b**,1). Superficial slides showing small areas of disorganization corresponding to the peripheral-side of dusty core (**b**,2), leading to a possible misdiagnosis with minicores. Deeper analysis revealing the real size of disorganization (**b**,3)

In all cases of DuCD, dusty cores were consistently associated to smaller areas of sarcomeric disorganization, but surprisingly none of our cases filled the morphological criteria to be classified as MmD. No muscle biopsy presented a multiminicore appearance by optic microscopy, and ultrastructural analysis revealed the presence of larger areas of disorganization. A possible explication of this finding could be that some small areas of disorganization could correspond to the peripheral-side of dusty cores (Fig. 6). Previously reported cases of MmD in *RYR1*-recessive myopathies, had very similar oxidative stains to those observed in DuCD [11, 18]. Furthermore, it has been elucidated that morphological features of *RYR1*-recessive MmD are quite different to those of *SEPN*-related MmD [13]. In particular, the previously reported cases of *RYR1*-recessive MmD were associated to type1 fibre predominance/uniformity, nuclear centralization and multiple large cores, as observed in our patients, whereas the *SEPN*-related MmD presents typically smaller and multiple boundless cores (“minicores”) [13].

Most of DuCD patients of this study, had been previously interpreted and classified as CNM with some atypical features (CNM “plus”) (Table 2) because, at that time, the internalisation of nuclei was considered the most relevant abnormality in spite of the areas of myofibrillar disorganisation and purple material deposition, since these areas of disorganization seem to appear later. The most frequent associated features were: irregular areas of myofibrillar network alteration, myofibrillar-like aggregates, or fuchsine material deposition. It is noteworthy that all these morphological features have not been reported in *BIN1, DNM2* or *MTM1*-related CNM [29]. Furthermore, dusty cores have never been detected in muscle biopsies of our series of CNM (7 BIN1, 29 DNM2, 32 MTM1). On the other hand, it’s worthy of note that a prominent nuclear internalization and centralization is a constant finding in all cases of DuCD, sometimes manifesting as the main histopathological feature. Nevertheless, also in these cases, a careful revision, consistently detected the presence of dusty cores in variable amount of muscle fibres, confirmed by EM study. Atypical morphological feature in DuCD group, without nuclear internalization or without type1 predominance, may be rarely observed.

Finally, dusty cores should be considered the unifying morphological feature among DuCD spectrum disorder and represent the morphological signature of *RYR1*-recessive myopathy, even when detected in only few fibres.

Few patients had been previously reported in the first paper of Bevilacqua et al. [4] in which muscle biopsies shared histopathological lesions consistent with dusty cores. At that time, we were not aware that these lesions could represent a hallmark of *RYR1*-recessive myopathy, as 7 cases represented a small cohort and it could had been an atypical variability of findings in *RYR1*-recessive patients. Only the systematically revision of muscle biopsies in all *RYR1*-recessive cases, allows us to realise that more than 50% of cases had dusty cores (sometimes only in few muscle fibres) in muscle biopsy. For this reason, we considered appropriate to give a specific name (dusty cores) to these lesions, considering it a specific entity among core diseases (dusty core disease). In this context, we identified DuCD as a subgroup of congenital core myopathies and we re-classified many *RYR1*-recessive patients, from CNM (or other morphological diagnosis) to DuCD group.

Taken together, all these considerations, lead to suppose that the cases reported as MmD and CNM related to *RYR1*-recessive mutations, possibly represent specific variant of DuCD spectrum, in which dusty cores could be few, underestimated, or appeared later in life, as occurred in some of our patients. This speculation is also supported by the evidence that ocular involvement is almost systematically present in the DuCD group, as reported in cases with MmD and CNM-related *RYR1*-recessive myopathies [1, 25], and in most severe patients. Most of severe cases belonged to the DuCD group, even if not statistically significant.

Interestingly the lowest level of RyR1 expression was observed in the DuCD group. These findings suggest a close relation between the RyR1 production and clinicopathological consequences. DuCD represent the last end morphological spectrum of *RYR1*- related myopathies. Possibly, a severe *RYR1* haploinsufficiency, as observed in DuCD with respect to the non-DuCD groups, could impair the stability and integrity of excitation-contraction coupling at triads level. Triads replication observed in several DuCD, could be the expression of tentative compensation of an insufficient RyR1 production. Indeed triads replication and T-tubule dilation has been observed also in dihydropyridine receptor (DHPR)-related congenital myopathy [34], which is the voltage-gated L-type Ca2+ channel located on the T-tubule-SR interface with RyR1.

As expected, the lowest level of RyR1 production is also associated to the most severe and early onset (<1 year) cases. The presence and stability of RyR1 protein is probably necessary not only for the sarcomeric structure maintenance, but also for the overall muscle function via efficient excitation-contraction coupling.

A last morphological consideration must be done with respect to the T1P+ group. Interestingly ocular involvement and moderate clinical presentation were frequent in T1P+. These findings could suggest a possible relation between T1P+ and DuCD, supporting the idea that T1P+ could represent an early-state of DuCD. This hypothesis is also supported by the observation of two patients (p12, p13) with the first muscle biopsy showing central nuclei with type1 uniformity and atrophy and the second one showing DuCD, suggesting an evolution of the myopathological lesions with the age. Moreover, all T1P+ cases had also some degree of sarcomeric disorganization at muscle biopsy. Nevertheless, the high level of RyR1 expression in T1P+ group makes this hypothesis controversial and leads to consider T1P+ group a separate entity. More data are warranted to confirm or refuse this hypothesis, as only 2 muscle biopsies of T1P+ group were available for the RyR1 expression study.

Genetic data in our cohort confirmed the high prevalence of missense variant in BSol domain in recessive forms of disease, even if lower than previously published data (40.5% vs 84%) [8]. Even if variants located in the BSol domain and the pore domain were more frequent in DuCD patients, no significant correlation has been found between the localization of variants and morphology. Finally, the RyR1 expression study leads to re-classify a number of genetic variants according to ACMG as follow: five class 3 “variant of unknown signification” (P4501L, Y4796H, R2458C, R1679H, M2120T) to class 4 (“probably pathogenic”) and four class 4 variants (M4875T, V1207M, R3903Q, E4911K) to class 5 (“pathogenic variants”). Nevertheless, *RYR1* haploinsufficiency might not be the only molecular mechanism to explain the pathophysiology: functional studies (e.g. calcium imaging test) could be helpful to evaluate the impact of each variant on RyR1 pathophysiology even if these approaches are too complex to be tested in routine diagnosis.

Finally, our results represent an emerging evidence in RYR1-recessive myopathies, but restricted to a specific study population, restricted in a monocentric study. More evidences are warranted to support our findings worldwide in other study populations and other laboratories.

## Conclusions

In conclusion, dusty core is the most frequent histopathological presentation of *RYR1*-recessive myopathies. Dusty cores are the unifying morphological lesion among the DuCD spectrum pathology and represent the morphological hallmark for the recessive form of disease. DuCD is associated to earlier disease onset, severe clinical phenotype and lowest *RYR1* expression in muscle.

## Additional file

Additional file 1: Full genetic data. (XLSX 470 kb)
